# Memory B cell subsets and plasmablasts are lower in early than in long-standing Rheumatoid Arthritis

**DOI:** 10.1186/s12865-014-0028-1

**Published:** 2014-09-04

**Authors:** Anna Laura Fedele, Barbara Tolusso, Elisa Gremese, Silvia Laura Bosello, Angela Carbonella, Silvia Canestri, Gianfranco Ferraccioli

**Affiliations:** Division of Rheumatology, Institute of Rheumatology and Affine Sciences, Catholic University of the Sacred Heart, Via Moscati 31, 00168 Rome, Italy

**Keywords:** Rheumatoid arthritis, B cells, ZAP-70, Autoantibodies, IL-6, BAFF

## Abstract

**Background:**

Alterations of B cell subset distribution have been described in the peripheral blood (PB) of rheumatoid arthritis (RA) patients, but no data are available on differences between the onset and the established phases of the disease. The purpose of the study was to clarify whether a peculiar distribution of B cell subsets characterizes RA onset, thus leading to a more favorable clinical response to treatment, and to evaluate the possible association of a particular B cell subpopulation with response to therapy.

**Results:**

122 RA patients were enrolled: 25 had symptom duration less than 3 months and were defined as having “very early RA” (VERA), and 43 had symptom duration from more than 3 months up to one year (early-RA: ERA). The other 54 RA patients had long-standing RA (LSRA). At baseline and at 6-month follow-up visit peripheral blood samples were collected and analyzed by flow cytometry for the distribution of circulating B cell subsets by staining with surface markers CD45, CD19, CD38, CD27 and IgD and intracellular marker ZAP70.

VERA and ERA patients showed higher percentages and absolute counts of circulating antigen inexperienced naïve B cells (IgD + CD27-) and lower percentages and absolute numbers of double negative (IgD-CD27-) memory B cells and plasmablasts (CD38 + CD27+) compared to LSRA patients. At the multivariate analysis, a higher frequency of naïve B cells (IgD + CD27-) at baseline arose as significant predictor of CDAI remission, together with “having VERA disease” and a low disease activity at baseline.

**Conclusions:**

The onset of RA is characterized by higher percentages and absolute numbers of naïve B cells and lower numbers of plasmablasts and double negative memory B cells compared to established RA. Naïve B cells could represent a promising biomarker of outcome.

**Electronic supplementary material:**

The online version of this article (doi:10.1186/s12865-014-0028-1) contains supplementary material, which is available to authorized users.

## Background

In the last years, B cells have been shown to play a key role in the pathogenesis of rheumatoid arthritis (RA), as suggested by rheumatoid factor (RF) and anti-citrullinated peptide antibody (ACPA) positivity as marker of disease severity that may precede the onset of symptoms by many years [1–4]; moreover, B cell depletion therapy has proven to be effective in seropositive patients [5]. Besides the production of autoantibodies, B cells act as antigen-presenting cells and secrete pro-inflammatory cytokines, such as interleukin-6 (IL-6), tumor necrosis factor (TNF) and interleukin-1 [6,7].

The elevation of B cell activation markers, such as beta2-microglobulin, immunoglobulin levels, free light chains of immunoglobulins and B-cell activating factor (BAFF) at the onset of RA highlights that B cells are involved in the early pathogenetic phases of the disease [8,9].

Alterations of B cell subset distribution have been described in the peripheral blood (PB) of RA patients. In long-standing RA (LSRA), an increased frequency of post-switch IgD-CD27+ memory B cells was found [10,11], while a lower frequency of pre-switch IgD + CD27+ memory B cells was observed, which normalized after anti-TNF therapy [11].

To date, few data are available regarding the B cell subset distribution at the beginning of RA. It is well known that a clinical “window of opportunity” exists (<12 weeks symptoms) for RA, since an early intervention may alter the propensity to persistence and therefore offer the opportunity of reaching remission [12]. There is a great need for biomarkers that can help the physicians to understand the pathogenetic mechanisms underlying this “therapeutic window”. Among these biomarkers, B cell subsets may be key drivers involved in the early phases of the disease.

Recently, a lower percentage of circulating total memory B cells and a significant decrease in the frequency of pre-switch antigen experienced memory B cells (IgD + CD27+) were described in RA patients with a disease duration less than six weeks as compared with controls [13]. No data are available on plasmablasts or double negative memory B cells.

Besides studying B cell surface markers, it is interesting to evaluate whether there is an imbalance in the intracellular activation markers of B lymphocytes, such as Zeta-chain-associated protein kinase 70 (ZAP-70). ZAP-70 is a protein kinase that is directly involved in the T cell signaling. Moreover, ZAP-70 has been shown to be expressed in some activated B cell subsets. We have demonstrated that B cells from the synovial compartment of RA patients express higher levels of ZAP-70 compared to B cells from patients with osteoarthritis and positively correlate with the synovial levels of autoantibodies, IL-6 and BAFF [14]. The functional significance of ZAP-70 in B-cell activation remains poorly documented. Recently, it has been proposed that B cell migration in vivo through the inflammatory sites is directly linked to a ZAP-dependent pathway mediated by the CXCR4/CD74 axis [15].

The aim of the study was to test the hypothesis that a different distribution of circulating memory B cells, plasmablasts and B cell activated subsets (i.e. CD19+/ZAP-70+ B cells) in early compared to long-standing RA could lead to a more favorable clinical response to treatment, and to evaluate whether the distribution of B cell subpopulations correlates with disease activity, particularly with the inflammatory milieu, at the onset of the disease. Finally, we examined whether B cell subset distribution at baseline could predict, as a biomarker, the clinical response at 24 weeks of follow-up in a treat to target strategy, and could be modified by conventional treatment in patients with RA at onset.

## Methods

### Patients

Peripheral blood samples from 122 RA patients and 30 healthy subjects, matched for age and sex, were collected for flow cytometric analysis. The ethical approval for the study was obtained from the Catholic University of the Sacred Heart Ethical Committee and all participants gave informed consent.

All RA patients fulfilled the 1987 and 2010 American College of Rheumatology (ACR) criteria for RA [16,17].

Sixty-eight of the 122 RA patients had symptom duration less than 12 months, of which 25 (36.8%) subjects with symptom duration less than 3 months were defined as having “very early RA (VERA)” [18]. The remaining 43 patients had symptom duration from more than 3 months up to one year (early-RA: ERA). Both VERA and ERA patients were naïve to disease-modifying antirheumatic drugs (DMARDs) and/or anti-TNF drugs at the time of enrollment.

VERA and ERA patients were followed every month up to three months and every three months thereafter. At each visit the ACR/ European League Against Rheumatism (EULAR) core data set (erythrocyte sedimentation rate (ESR), C-reactive protein (CRP), swollen joint count (SJC), tender joint count (TJC), physician and patient global assessment, pain, health assessment questionnaire (HAQ)) was registered and DAS was calculated [19].

At baseline, VERA and ERA patients were treated with methotrexate (up to 20 mg weekly) and, when necessary, with a low steroid dose for three months; then a combination with a TNF blocker (adalimumab 40 mg every two weeks, or etanercept 50 mg weekly) was started if patients did not reach at least a good response according to EULAR criteria (DAS44: DAS ≤2.4) [20]. At each visit, clinical improvement and remission were evaluated according to DAS [20,21] and CDAI cut-off points [22].

The other 54 RA patients had a long-standing disease (LSRA; mean disease duration of 12.5 ± 7.9 years). LSRA patients were consecutively selected from RA out-patients who had an active disease (DAS > 2.4) despite being on treatment with one or more DMARDs and/or anti-TNF drugs.

### Flow cytometry and immunophenotyping of B cells

PB samples were collected at baseline from 122 RA patients and at 6-month follow-up visit from 61 RA patients and immunofluorescence labelling for flow cytometric analysis of circulating B cell subpopulations was performed by incubating 100 μl of PB with anti-human monoclonal antibodies. Cells were stained with anti-human antibodies specific for CD45 (APC-A750), CD19 (APC-700), CD3 (ECD), CD56 (ECD), CD38 (PC5 or APC), CD27 (PC7) and FITC-conjugated IgD (Beckman Coulter, Marseille France). After 20 minutes of incubation, the samples were fixed with 200 μl of Reagent 1 (Beckman Coulter, Marseilles, France) for 15 minutes at room temperature (RT) in the dark. After washing, the pellet was incubated with 200 μl of Reagent 2 (Beckman Coulter, Marceilles, France) for 15 minutes at RT in the dark. The samples were then incubated with PE-conjugated ZAP-70 specific AB (clone SBZAP, Beckman Coulter, Marseilles, France) for 30 minutes at RT in the dark. After staining, the cells were washed and immediately analyzed on properly compensated 8 colors Navios flow-cytometer and data were elaborated with the Kaluza program (Beckman Coulter, Marseille, France). Lymphocytes were gated on the basis of forward-and side-scatter properties (confirmed through CD45 staining) and at least 10,000 CD19+ cells were analyzed. B cell subsets were evaluated through the expression of surface B cell markers according to IgD/CD27 classification [23].

The expression of ZAP-70 on CD19+ cells was measured according to the gating strategy published by Crespo et al. [24]. B cells were defined as CD56 and CD3 negative and CD19 positive cells. The percentage of ZAP-70 positive B cells was evaluated at 98% of ZAP-70 positivity of T-NK cells [25].

### Detection of Autoantibodies

RF-IgM and RF-IgA (Orgentec Diagnostika GmbH, Mainz, Germany), ACPA (Axis Shield Diagnostics, Dundee, UK) and anti-mutated citrullinated vimentin (MCV) (Orgentec Diagnostika, Dundee, UK) were measured using commercial ELISA and performed according to the manufacturer’s instruction. The suggested cut-off levels were 20 U/ml for RF-IgM, RF-IgA and anti-MCV and 5 U/ml for ACPA.

### Soluble biomarkers

Blood samples were collected from all patients at baseline and from VERA and ERA patients six months after starting therapy. Samples were immediately centrifuged and stored at −80°C until analysis. Plasma levels of IL-6 and BAFF were measured by ELISA (R&D Systems, UK). The sensitivity of the test was of 2.2 pg/ml for IL-6 and 2.4 pg/ml for BAFF.

### Statistical analysis

Statistical analysis was performed using SPSS (SPSS version 16.0, Chicago, IL, USA) and Graph-Pad Prism statistical software (San Diego, CA, USA). Categorical and quantitative variables were recorded as frequencies, percentage, mean ± Standard Deviation (SD). The non-parametric Mann–Whitney U test was used to compare the continuous variables. Categorical variables were analyzed using χ^2^ test or the Fisher’s exact test.

The Spearman rank correlation was used to evaluate the relationship between the different B cell subsets and between B cell subsets and inflammatory and clinical parameters and the Wilcoxon test was used to compare the B cell subpopulations and soluble biomarkers during follow-up in the VERA and ERA cohort. A receiver operating characteristic (ROC) analysis of the continuous parameters related to the response to therapy was performed by plotting the relationship between sensitivity, on the y-axis, and 1-specificity, on the x-axis, for different cut-off levels of test positivity. The area under the ROC curve (ROC AUC) provides a measure of the overall discriminative ability of a model. The ROC area and its standard error (SE) were estimated using the nonparametric approach. The optimal cut-off point was determined to yield the maximum corresponding sensitivity and specificity.

The variables related to response to therapy in VERA and ERA patients with a p ≤ 0.15 at the univariate analysis entered into a multivariate logistic regression model in which “DAS remission or CDAI remission at 24 week follow-up visit” was the variable to be explained. Results are expressed as the odds ratio (OR) and 95% confidence interval (95% CI). Statistical significance was defined as p <0.05.

## Results

### Characteristics of the study populations

There were no differences in demographic, clinical and immunological parameters between patients with VERA, ERA and LSRA. Each cohort had a moderate-severe disease at study entry and no difference was seen between groups (DAS: 3.7 ± 1.1 in VERA, 3.3 ± 1.0 in ERA and 3.7 ± 1.4 in LSRA patients) (Table 1).

**Table 1:** **Demographic and clinical characteristics of RA patients (VERA, ERA and LSRA) at the study entry**

**Variables**	**VERA**	**ERA**	**LSRA**
**N.**	25	43	54
**Age,** years	57.5 ± 13.0	50.4 ± 14.9	53.0 ± 13.7
**Sex,** n. female, (%)	21 (84.0)	38 (88.4)	45 (86.5)
**ESR,** mm/1^^^hour	51.4 ± 33.7	39.1 ± 28.6	39.6 ± 23.8
**CRP,** mg/l	29.9 ± 45.5	16.2 ± 25.3	20.3 ± 28.0
**DAS**	3.7 ± 1.1	3.3 ± 1.0	3.7 ± 1.4
**ACPA positivity,** n. (%)	12 (48.0)	28 (65.1)	37 (72.5)
**RF-IgM positivity,** n. (%)	12 (48.0)	25 (58.1)	28 (54.9)
**RF-IgA positivity,** n. (%)	7 (28.0)	17 (39.5)	16 (31.4)

Values are mean ± standard deviation unless otherwise indicated. ERA: early rheumatoid arthritis; LSRA: long-standing rheumatoid arthritis; VERA: very early rheumatoid arthritis; ESR: erythrocyte sedimentation rate; CRP: C-reactive protein; DAS: disease activity score; ACPA: anti-citrullinated peptide antibodies; RF: rheumatoid factor.

At study entry, 4 VERA (16.0%) and 9 ERA (20.9%) patients had already been treated with low doses of corticosteroids (less than 10 mg of prednisone daily). Among patients with LSRA, 24 (44.4%) were taking DMARDs, while a combination therapy with anti-TNF was administered to 30 (55.6%) of them.

### B lymphocyte frequency is different according to RA disease duration

VERA and ERA patients showed similar percentages of CD19+ cells (VERA: 10.1 ± 4.5%; ERA: 9.8 ± 4.2%), gated on total lymphocytes, with controls (9.5 ± 2.6%) but significantly higher compared to LSRA patients (6.9 ± 4.2%, p = 0.002 vs VERA and p = 0.001 vs ERA). There were no differences in the absolute B cell number between the three analyzed cohorts (data not shown).

### Lower frequency and absolute count of double negative memory B cells and plasmablasts in VERA and ERA compared to LSRA patients

We analyzed the main B cell subsets, according to their IgD and CD27 expression [23]. The frequency and total count number of antigen inexperienced naïve B cells (IgD + CD27-), antigen experienced pre-switched memory B cells (IgD + CD27+), post-switched memory B cells (IgD-CD27+), double negative memory B cells (IgD-CD27-) and CD27 + CD38+ B cells from VERA and ERA patients were compared to LSRA patients.

**Figure 1:**
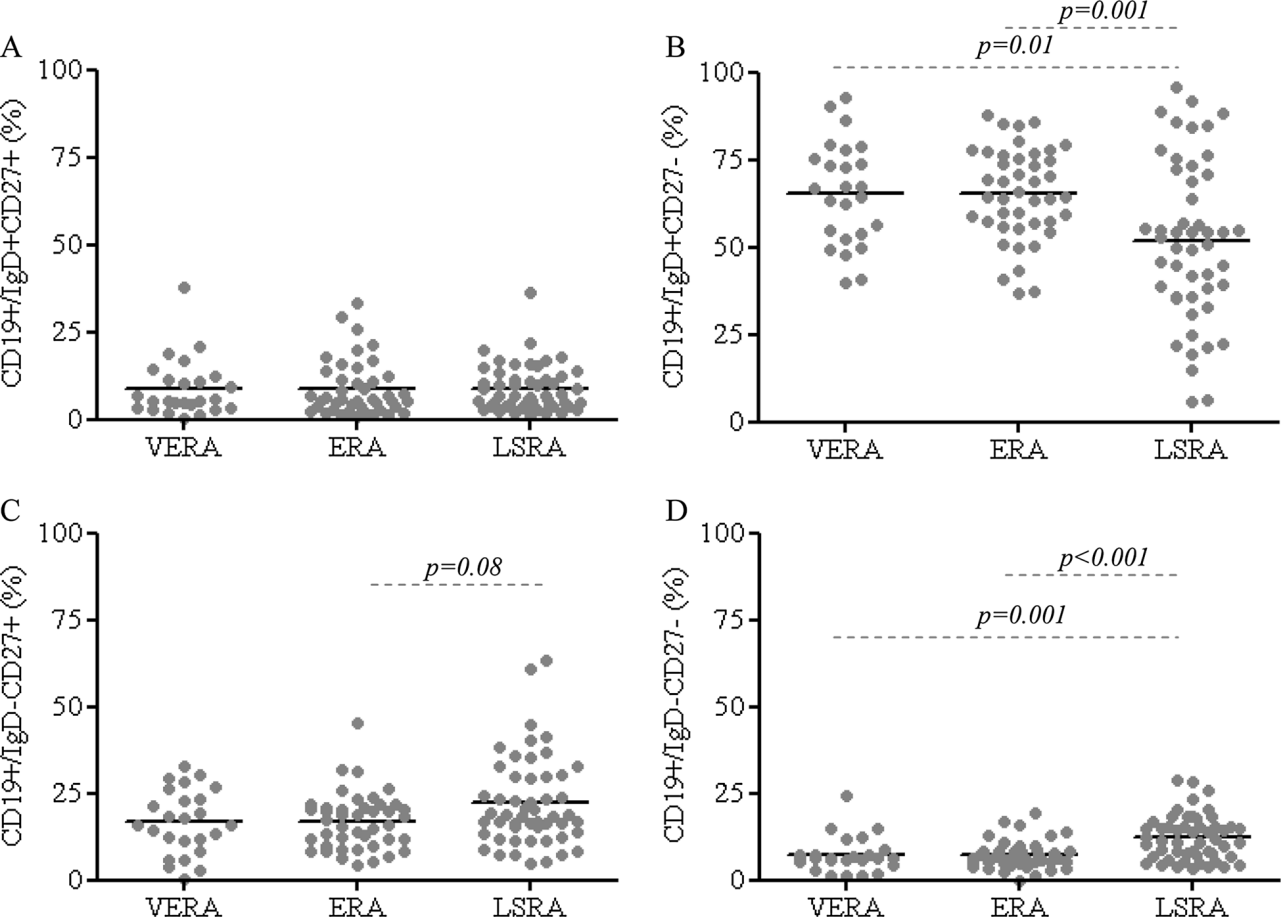
**Distribution of the percentage of the different B cell subsets in patients with VERA, ERA and LSRA patients, according to the IgD and CD27 classification.** Plots represent the percentage of IgD + CD27+ cells **(A)**, IgD + CD27- cells **(B)**, IgD-CD27+ cells **(C)** and IgD-CD27- cells **(D)**.

**Figure 2:**
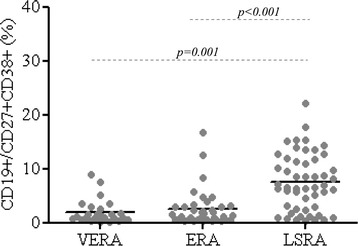
**Percentage of circulating CD38 + CD27+ B cells in VERA, ERA RA and LSRA patients.**

Similar percentages and absolute count of B cell subsets were observed in VERA and ERA patients (Figure 1, Additional file 1: Table S1 and Additional file 2: Figure S1) and controls. The naïve B cell subset (IgD + CD27-) (both percentage and absolute count) was increased significantly in both VERA and ERA patients compared to patients with LSRA, whereas the percentage and absolute count of IgD-CD27- B cells was lower in VERA and ERA patients compared to LSRA (Figure 1B and 1D, Additional file 1: Table S1 and Additional file 2: Figure S1). Moreover, the frequency and number of CD19+/CD27 + CD38+ cells were lower in VERA and ERA patients compared to LSRA patients (Figure 2, Additional file 1: Table S1 and Additional file 2: Figure S1).

These data suggest that the early phases of RA are characterized by an expansion of naïve-activated B cell subset and a lower frequency and number of double negative memory B cells and plasmablasts compared to the later stages of the disease.

No differences were found in B cell subset distribution dividing patients according to demographic parameters, positivity for ACPA or RF, both IgM and IgA, and steroid therapy in VERA and ERA patients at baseline.

Finally, the B cell subset distribution was similar according to the different therapeutic strategies in LSRA patients (data not shown).

### Plasma BAFF and IL-6 levels differ between RA at onset and established RA

**Figure 3:**
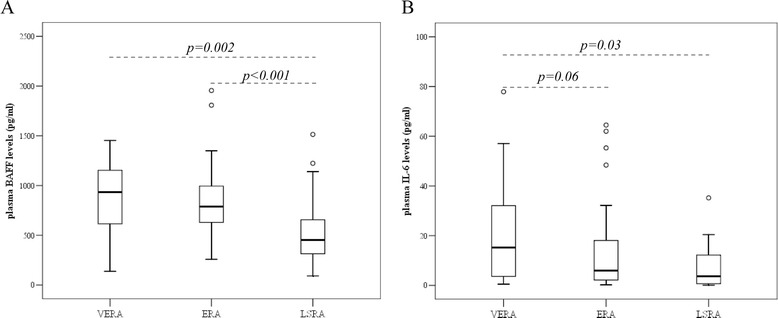
**Plasma BAFF (A) and IL-6 (B) levels in VERA, ERA and LSRA patients.** The Mann–Whitney test was used for comparison of unpaired data.

Plasma BAFF levels were significantly higher in both VERA (p = 0.002) and ERA patients (p < 0.001) compared to LSRA patients (Figure 3A), whereas plasma IL-6 levels where higher in the VERA cohort when compared to LSRA subjects (*p = 0.03*) (Figure 3B). No difference was seen in plasma BAFF and IL-6 levels between VERA and ERA patients.

In VERA and ERA patients, plasma BAFF and IL-6 levels at baseline were not influenced by demographic parameters and corticosteroid use. Moreover, plasma levels of IL-6 and BAFF were similar in the subgroup of LSRA, independently from the different therapeutic strategies (data not shown).

### Correlations between plasma BAFF and IL-6 levels and B cell subsets in VERA and ERA patients

In VERA and ERA patients, plasma BAFF levels directly correlated with naïve B cell (IgD + CD27-, r = 0.31, *p = 0.01*), plasmablast (r = 0.26, *p = 0.05*) and memory IgD-CD27- B cell (r = 0.31, *p = 0.01*) frequencies, and inversely correlated with unswitched- (IgD + CD27+: r = 0.41, *p = 0.001*) and switched- (IgD-CD27+: r = 0.33, *p = 0.01*) memory B cell frequencies.

There were no significant correlations between plasma IL-6 levels and the different B cell subsets, in the three cohorts of RA patients (data not shown).

### ZAP-70 expression in B cells of VERA, ERA and LSRA patients

The percentage and absolute number of B cells expressing intracellular ZAP-70 were similar in the three analyzed cohorts of RA patients (Additional file 1: Table S1), but higher than in healthy controls (1.8 ± 0.9% , p = 0.001 vs VERA, p = 0.002 vs ERA and p = 0.04 vs LSRA).

As noted in an our recent work [25], ZAP-70+ B cells show a phenotype belonging to the memory pool [IgD + CD27+ (p = 0.003), IgD-CD27+ (p < 0.001) and IgD-CD27- (p < 0.001)] when compared to the ZAP-70 negative ones (Additional file 3: Figure S2).

As expected, the percentage of circulating ZAP-70+ B cells directly correlated with the percentages of plasmablasts (r = 0.32, *p = 0.01*) and of double negative memory B cells (r = 0.37, *p = 0.002*), in VERA and ERA patients.

Finally, both the percentage (r = 0.40, *p = 0.002*) and the number (r = 0.35, *p = 0.01*) of circulating CD19+/ZAP-70+ cells directly correlated with plasma BAFF levels.

### Disease activity and B cell subset distribution in VERA and ERA patients at baseline

VERA and ERA patients with a severe disease at baseline (DAS >3.7) showed lower percentages of CD19+ cells (8.4 ± 4.0%) compared to subjects with a low-moderate disease activity (DAS < 3.7) (11.2 ± 4.4%, *p = 0.02*) and higher percentages and numbers of CD19+/CD27 + CD38+ (CD19+/CD27 + CD38+ (%): 4.4 ± 5.9% *vs* 1.2 ± 1.1%, *p = 0.004* and CD19+/CD27 + CD38+ cells/μl: 7.2 ± 15.2 vs 3.2 ± 4.7 cells/μl; p = 0.04). We found no correlation between disease activity at baseline and the other B cell subsets, both in number and percentage (data not shown).

### Baseline predictive factors associated with response to therapy at 24 week follow-up visit in VERA and ERA patients

Forty-five out of 61 VERA and ERA patients (73.8%) were classified as good EULAR responders, while 32 (52.5%) were in DAS remission (DAS < 1.6) and 20 (32.8%) in CDAI-remission (CDAI ≤ 2.8) at 24 week follow-up visit. Seven VERA and ERA patients were lost during follow-up.

At 24 weeks of follow-up, 17 (27.9%) patients were in combination therapy with anti-TNF drugs, while the other 44 patients were in monotherapy with DMARDs (72.1%). The remission rate was similar in patients treated with DMARDs (DAS remission: 50.0%, CDAI remission: 27.3%) and anti-TNF drugs (DAS remission: 58.8%, *p = 0.54;* CDAI remission: 47.1%, *p = 0.14*) at 24 week follow-up visit.

The baseline disease characteristics and the B cell subset distribution were, first, analyzed in correlation with DAS remission at 24 weeks. By univariate analysis, younger age, lower inflammatory markers and lower disease activity, a higher frequency of naïve B cells (IgD + CD27-) and a lower frequency of switched-memory B cells (IgD-CD27+) were associated with DAS remission and subsequently included in the multivariate analysis. At the multivariate analysis, a younger age at diagnosis (OR(95% CI): 0.92 (0.87-0.97)) and a higher frequency of naïve B cells (IgD + CD27-) (OR(95% CI): 1.06 (1.01-1.11)) were significantly associated with DAS remission at 24 weeks of follow-up (Table 2). A more stringent definition of remission (i.e. CDAI remission) was evaluated in the same cohort of patients. At the multivariate analysis, “having VERA disease” (OR(95% CI): 8.89 (1.79-44.20)) and a low disease activity at baseline (OR(95% CI): 0.34 (0.14-0.83)) arose as significant predictors of CDAI remission together with a higher frequency of naïve B cells (IgD + CD27-) at baseline (OR(95% CI): 1.09 (1.02-1.17)).

**Table 2:** **Baseline demographic, clinical and immunological characteristics and B cell subset distribution (IgD and CD27 classification) in VERA and ERA patients according to response to therapy (i.e. remission) at 24 weeks of follow-up**

**Variables**	**DAS remission**	**No DAS remission**	**Univariate analysis; p value**	**Multivariate analysis; OR (95% CI)**	**CDAI remission**	**No CDAI remission**	**Univariate analysis; p value**	**Multivariate analysis; OR (95% CI)**
**N.**	32	29			20	41		
**Age,** years	47.0 ± 15.2	61.5 ± 10.3	***<0.001***	**0.92 (0.87-0.97)**	50.2 ± 15.7	55.7 ± 14.3	*0.21*	
**Sex,** n. female, n. (%)	26 (81.3)	26 (89.7)	*0.36*		16 (80.0)	36 (87.8)	*0.42*	
**VERA,** n. (%)	13 (40.6)	9 (31.0)	*0.44*		11 (55.0)	11 (26.8)	***0.03***	**8.89 (1.79-44.2)**
**Anti-TNF therapy**	10 (31.3)	6 (20.7)	*0.40*		8 (40.0)	8 (19.5)	*0.09*	
**ESR,** mm/1^^^hour	33.6 ± 23.2	54.0 ± 35.2	***0.02***	1.00 (0.96-1.04)	41.5 ± 30.3	54.0 ± 35.2	*0.58*	
**CRP,** mg/l	11.3 ± 18.2	32.7 ± 45.9	***0.01***	0.98 (0.95-1.01)	18.7 ± 36.8	32.7 ± 45.9	*0.23*	
**DAS**	3.1 ± 1.0	3.8 ± 1.1	***0.05***	0.58 (0.21-1.57)	3.2 ± 0.9	3.6 ± 1.1	*0.13*	**0.34 (0.14-0.83)**
**CDAI**	24.3 ± 12.4	28.7 ± 14.3	*0.36*		23.7 ± 11.4	27.7 ± 14.3	*0.32*	
**ACPA positivity,** n. (%)	23 (71.9)	15 (51.7)	*0.11*	2.21 (0.57-8.66)	16 (80.0)	22 (53.7)	***0.05***	2.05 (0.36-11.6)
**RF-IgM positivity,** n. (%)	20 (62.5)	15 (51.7)	*0.40*		14 (70.0)	21 (51.2)	*0.16*	
**RF-IgA positivity,** n. (%)	12 (37.5)	11 (37.9)	*0.97*		8 (40.0)	15 (36.6)	*0.80*	
**BAFF, pg/ml**	878.3 ± 430.2	819.7 ± 265.3	*0.77*		991.0 ± 484.3	780.8 ± 264.1	*0.08*	1.002 (1.000-1.004)
**IL6 (pg/ml)**	13.4 ± 20.4	22.3 ± 24.8	***0.02***	1.02 (0.98-1.06)	18.0 ± 24.0	17.5 ± 22.5	*0.79*	
**CD19+/IgD + CD27+,** %	8.3 ± 8.2	13.7 ± 18.7	*0.25*		7.3 ± 8.3	12.6 ± 16.3	*0.08*	1.03 (0.90-1.17)
**CD19+/IgD + CD27-,** %	67.5 ± 13.6	57.0 ± 17.2	***0.01***	**1.06 (1.01-1.11)**	68.7 ± 11.6	59.5 ± 17.3	***0.03***	**1.09 (1.02-1.17)**
**CD19+/IgD-CD27+,** %	15.2 ± 7.7	19.3 ± 8.8	*0.08*	1.06 (0.92-1.22)	14.6 ± 7.7	18.4 ± 8.5	*0.09*	1.09 (0.88-1.33)
**CD19+/IgD-CD27-,** %	7.2 ± 4.7	7.4 ± 4.2	*0.58*		7.7 ± 5.0	7.1 ± 4.2	*0.47*	
**CD19+/CD38 + CD27+,** %	2.0 ± 2.1	3.1 ± 5.4	*0.84*		1.8 ± 1.4	2.9 ± 4.8	*0.94*	
**CD19+/ZAP70+,** %	5.9 ± 7.4	4.1 ± 3.3	*0.86*		6.5 ± 8.1	4.3 ± 4.4	*0.18*	

Values are mean ± standard deviation unless otherwise indicated. The values in bold are those that arose as significant in univariate and multivariate analyses. ERA: early rheumatoid arthritis; LSRA: long-standing rheumatoid arthritis; VERA: very early rheumatoid arthritis; ESR: erythrocyte sedimentation rate; CRP: C-reactive protein; DAS: disease activity score; ACPA: anti-citrullinated peptide antibodies; RF: rheumatoid factor.

When DMARD users were selected (n = 44), “having VERA disease” (OR(95% CI): 8.05 (1.16-55.99)) together with a higher frequency of naïve B cells (IgD + CD27-) at baseline (OR(95% CI): 1.09 (1.01-1.17)) were confirmed as the independent variables associated with CDAI remission.

We next performed ROC analyses for the circulating percentage of naïve B cells (IgD + CD27-) to predict remission (both DAS remission and CDAI remission) at the 24 weeks follow-up. The areas under the ROC curve (AUCs) were 0.743 ± 0.074 for DAS remission and 0.695 ± 0.092 for CDAI remission, which are at the fair to good threshold. Considering the CDAI remission, the sensitivity and specificity (cut-off value: 60.0%) were 75.0% and 53.0%, respectively.

Patients with circulating IgD + CD27- B cells at baseline > 60.0% had 4 times higher chances of reaching remission than patients with lower percentages [(OR(95% CIs): 4.2 (1.2-14.7)].

The multivariate analysis was not performed in the subgroup of anti-TNF treated patients due to the limited number of cases (n = 17).

### Influence of conventional therapy on clinical and inflammatory parameters and B cell subset distribution over time in VERA and ERA patients

Considering the subgroup of VERA and ERA patients treated with DMARDs, a reduction of the inflammatory biomarkers and disease activity was observed at 24 week follow-up visit, together with a significant decrease of RF-IgM and RF-IgA titers (Additional file 4: Table S2). No difference was seen in B cell subsets over time (both percentage and absolute number), both in patients reaching remission, defined as DAS < 1.6 or CDAI ≤ 2.8 and in those not responding to therapy. The low number of patients receiving anti-TNF agents forbids a definite conclusion, even though treatment with anti-TNF seemed to reduce the percentage of CD19+/ZAP70+ cells at 24 weeks (8.4 ± 9.5% at baseline vs 3.7 ± 2.6% at 24 weeks, p = 0.04) in VERA and ERA patients (n = 17).

## Discussion

To date few data are available regarding the distribution of circulating B cell subsets in the early phases of RA. The aim of our study was to examine whether there could be a peculiar B cell subset distribution in the PB of patients with RA at onset compared to long-standing RA and to evaluate a possible association of B cell subpopulations with response to therapy.

In this study, we observed significant differences in the distribution of circulating B cell subsets, both in absolute number and percentage, between VERA and ERA compared to LSRA patients. Analyzing in detail the different B cell subsets, VERA and ERA patients showed higher percentages and numbers of antigen inexperienced naïve IgD + CD27- B cells compared to LSRA patients, and lower percentages and numbers of IgD and CD27 negative B cells and of plasmablasts (CD38 + CD27+).

The IgD + CD27- naïve B cells have been identified as those likely containing auto-reactive B cells and carrying predominantly auto-reactive B cell receptors [26]. Therefore, it appears clear that the predominance of naïve B cells in the early phases of the disease is biologically relevant, particularly when devising a possible therapeutic strategy, since recently it has been shown *in vitro* that Rituximab, a chimeric monoclonal antibody that recognizes human CD20, inhibited the proliferation of CD27- naïve, but not of CD27+ memory B cells [27].

Our results partially confirm recently published data by Moura et al. on B cell subset distribution in a small cohort of RA patients with a disease duration less than six weeks. These authors observed lower percentages of total memory B cells and higher percentages of naïve B cells compared to controls [13].

In addition, our data suggest that the B cell biology is strictly similar in VERA and ERA and that plasmablast frequency is a marker of disease activity.

When RA patients with a long-standing disease were examined, higher percentages of switched-memory B cells were observed and a strong correlation of this subset with disease duration was found [10]. It has been hypothesized that in LSRA patients the memory B cells accumulated in the synovial compartment, probably under the influence of TNF [10,11].

The lower percentage of circulating memory B cells observed in VERA and ERA patients compared to LSRA could be explained by the possible segregation of these cells in the synovial compartment. The increase of naïve activated cells could be reactive to the recruitment of these cells in ectopic lymphoid tissue. The sequestration of memory B cells in the synovium has been demonstrated, recently, by our group, which found higher percentages of IgD-CD27+ and IgD-CD27- B cells and lower percentages of naïve B cells in the synovial fluid compared to the PB of patients with RA [25].

Interestingly, in this study, we found a lower percentage of memory B cells double negative for IgD and CD27 in VERA and ERA compared to LSRA patients. This cell subset is expanded in patients with systemic lupus erythematosus (SLE), in which it correlates significantly with disease activity [28]. Therefore, we can hypothesize that a major involvement of this B cell specific subpopulation could characterize the progression of RA and the more severe forms of the disease, as it happens in SLE.

A possible clue to understand the compartmentalization of B cell subsets could be related to the cytokine milieu characteristic of the early phases of RA. The onset of the disease is characterized by higher plasma levels of BAFF and IL-6 compared to controls, as it has already been reported [8,9,29,30].

The higher plasma IL-6 levels observed in patients with VERA, support experimental data which describe this cytokine among those that increase earlier and that guide the transition from acute to chronic inflammation [31]. Moreover, the early phases of the disease were characterized by higher plasma BAFF levels compared to LSRA patients, which positively correlate with the percentage of plasmablasts, of double negative memory B cells and of B lymphocytes positive for ZAP-70, a possible biomarker of B cell activation.

Our group recently described higher percentages of ZAP-70 positive B cells, exhibiting a memory/plasmablast phenotype, mostly in the aggregate pattern of synovial infiltrates and its value as a biomarker of activity and efficacy in RA patients treated with B-cell depletion therapy (BCDT) [25,32].

At the multivariate analysis, a younger age at diagnosis, “having VERA disease” and a higher frequency of naïve B cells were significantly associated with remission at 24 weeks of follow-up. These results confirmed our previous data that VERA represents the best opportunity to reach complete disease remission [12]. An interesting finding of our study is that a higher percentage of the naïve activated B cell subset (i.e. IgD + CD27-) at baseline has emerged as a predictor of clinical remission. In particular, patients with RA at onset with high percentages of circulating naïve-activated B cells at baseline (>60.0%) were 4 times more likely to achieve CDAI remission after 24 weeks of treatment compared to patients with a low percentage of CD19+/IgD + CD27-.

Finally, the B-cell subsets were not affected by DMARD therapy, thus raising the issue of which type of remission is achieved. A limit of our study is the small size of patients treated with anti-TNF that not allow to assess whether this therapy influences the distribution of B cell subsets over time.

## Conclusions

In conclusion, the onset of RA is characterized by higher percentages and absolute counts of naïve-activated B cells and lower percentages and numbers of double negative memory B cells and of plasmablasts, mostly related to disease activity, compared to long-standing RA. This could represent a more favorable biological setting for therapies aiming at remission. The relationship between some B cell phenotypes and remission, strongly supports the rationale for studying activation and function of their different subsets.

## Additional files

Additional file 1: Table S1. B cell subset distribution according to IgD and CD27 classification in patients with VERA, ERA and LSRA. Additional file 2: Figure S1. Distribution of the absolute number of the different B cell subsets in patients with VERA, ERA and long standing-RA, according to the IgD and CD27 classification. Plots represent the absolute numbers of IgD + CD27+ cells (A), IgD + CD27- cells (B), IgD-CD27+ cells (C), IgD-CD27- cells (D), CD38 + CD27+ cells (E) and CD19+/ZAP-70+ (F) B cells. Additional file 3: Figure S2. Phenotypic characterization of ZAP-70+ and ZAP-70- B cells in VERA and ERA patients at baseline. The plot represents the percentage of IgD + CD27+ cells, IgD + CD27- cells, IgD-CD27+ and IgD-CD27- cells in CD19+/ZAP-70+ (grey) and in CD19+/ZAP-70- (white) cells. Box plot show the 10th, 25th, 50th (median), 75th and 90th percentiles of the variable. Additional file 4: Table S2. Clinical and immunological parameters in patients with VERA and ERA, treated with conventional therapy, at the study entry and at 24 weeks follow-up visit.

## Abbreviations

RA: Rheumatoid Arthritis
PB: Peripheral blood
LSRA: Long-standing RA
VERA: Very early RA
ERA: Early-RA
RF: Rheumatoid factor
ACPA: Anti-citrullinated peptide antibody
IL-6: Interleukin-6
TNF-α: Tumor necrosis factor-a
IL-1: Interleukin-1
BAFF: B-cell activating factor
ZAP-70: Zeta-chain-associated protein kinase 70
ESR: Erythrocyte sedimentation rate
CRP: C-reactive protein
SJC: Swollen joint count
TJC: Tender joint count
HAQ: Health assessment questionnaire
ACR: American College of Rheumatology
EULAR: European League Against Rheumatism
CDAI: Clinical Disease Activity Index
DMARDs: Disease-modifying antirheumatic drugs
anti-MCV: Anti-mutated citrullinated vimentin
ELISA: Enzyme-linked immunosorbent assay
ROC: Receiver operating characteristic
AUC: Area under the curve
OR: Odds ratio

## References

[1] Vencovský J, Machácek S, Sedová L, Kafková J, Gatterová J, Pesáková V, Růzicková S (2003). Autoantibodies can be prognostic markers of an erosive disease in early rheumatoid arthritis. Ann Rheum Dis.

[2] Machold KP, Stamm TA, Nell VP, Pflugbeil S, Aletaha D, Steiner G, Uffmann M, Smolen JS (2007). Very recent onset rheumatoid arthritis: clinical and serological patient characteristics associated with radiographic progression over the first years of disease. Rheumatology (Oxford).

[3] Nielen MM, van Schaardenburg D, Reesink HW, van de Stadt RJ, van der Horst-Bruinsma IE, de Koning MH, Habibuw MR, Vandenbroucke JP, Dijkmans BA (2004). Specific autoantibodies precede the symptoms of rheumatoid arthritis: a study of serial measurements in blood donors. Arthritis Rheum.

[4] Berglin E, Johansson T, Sundin U, Jidell E, Wadell G, Hallmans G, Rantapää-Dahlqvist S (2006). Radiological outcome in rheumatoid arthritis is predicted by presence of antibodies against cyclic citrullinated peptide before and at disease onset, and by IgA-RF at disease onset. Ann Rheum Dis.

[5] Chatzidionysiou K, Lie E, Nasonov E, Lukina G, Hetland ML, Tarp U, Gabay C, van Riel PL, Nordström DC, Gomez-Reino J, Pavelka K, Tomsic M, Kvien TK, van Vollenhoven RF (2011). Highest clinical effectiveness of rituximab in autoantibody-positive patients with rheumatoid arthritis and in those for whom no more than one previous TNF antagonist has failed: pooled data from 10 European registries. Ann Rheum Dis.

[6] Marston B, Palanichamy A, Anolik JH (2010). B cells in the pathogenesis and treatment of rheumatoid arthritis. Curr Opin Rheumatol.

[7] Silverman GJ, Boyle DL (2008). Understanding the mechanistic basis in rheumatoid arthritis for clinical response to anti-CD20 therapy: the B cell roadblock hypothesis. Immunol Rev.

[8] Gottenberg JE, Miceli-Richard C, Ducot B, Goupille P, Combe B, Mariette X (2009). Markers of B-lymphocyte activation are elevated in patients with early rheumatoid arthritis and correlated with disease activity in the ESPOIR cohort. Arthritis Res Ther.

[9] Bosello S, Youinou P, Daridon C, Tolusso B, Bendaoud B, Pietrapertosa D, Morelli A, Ferraccioli G (2008). Concentrations of BAFF correlate with autoantibody levels, clinical disease activity, and response to treatment in early rheumatoid arthritis. J Rheumatol.

[10] Fekete A, Soos L, Szekanecz Z, Szabo Z, Szodoray P, Barath S, Lakos G (2007). Disturbances in B- and T-cell homeostasis in rheumatoid arthritis: suggested relationships with antigen-driven immune responses. J Autoimmun.

[11] Souto-Carneiro MM, Mahadevan V, Takada K, Fritsch-Stork R, Nanki T, Brown M, Fleisher TA, Wilson M, Goldbach-Mansky R, Lipsky PE (2009). Alterations in peripheral blood memory B cells in patients with active rheumatoid arthritis are dependent on the action of tumour necrosis factor. Arthritis Res Ther.

[12] Gremese E, Salaffi F, Bosello SL, Ciapetti A, Bobbio-Pallavicini F, Caporali R, Ferraccioli G (2013). Very early rheumatoid arthritis as a predictor of remission: a multicentre real life prospective study. Ann Rheum Dis.

[13] Moura RA, Weinmann P, Pereira PA, Caetano-Lopes J, Canhão H, Sousa E, Mourão AF, Rodrigues AM, Queiroz MV, Souto-Carneiro MM, Graça L, Fonseca JE (2010). Alterations on peripheral blood B-cell subpopulations in very early arthritis patients. Rheumatology (Oxford).

[14] Tolusso B, De Santis M, Bosello S, Gremese E, Gobessi S, Cuoghi I, Totaro MC, Bigotti G, Rumi C, Efremov DG, Ferraccioli G (2009). Synovial B cells of rheumatoid arthritis express ZAP-70 which increases the survival and correlates with the inflammatory and autoimmune phenotype. Clin Immunol.

[15] Klasen C, Ohl K, Sternkopf M, Shachar I, Schmitz C, Heussen N (2014). MIF Promotes B Cell Chemotaxis through the Receptors CXCR4 and CD74 and ZAP-70 Signaling. J Immunol.

[16] Arnett FC, Edworthy SM, Bloch DA, McShane DJ, Fries JF, Cooper NS, Healey LA, Kaplan SR, Liang MH, Luthra HS, Medsger TA, Mitchell DM, Neustadt DH, Pinals RS, Schaller JG, Sharp JT, Wilder RL, Hunder GG (1988). The American Rheumatism Association 1987 revised criteria for the classification of rheumatoid arthritis. Arthritis Rheum.

[17] Aletaha D, Neogi T, Silman AJ, Funovits J, Felson DT, Bingham CO, Birnbaum NS, Burmester GR, Bykerk VP, Cohen MD, Combe B, Costenbader KH, Dougados M, Emery P, Ferraccioli G, Hazes JM, Hobbs K, Huizinga TW, Kavanaugh A, Kay J, Kvien TK, Laing T, Mease P, Ménard HA, Moreland LW, Naden RL, Pincus T, Smolen JS, Stanislawska-Biernat E, Symmons D (2010). 2010 Rheumatoid arthritis classification criteria: an American College of Rheumatology/European League Against Rheumatism collaborative initiative. Arthritis Rheum.

[18] Bosello S, Fedele AL, Peluso G, Gremese E, Tolusso B, Ferraccioli G (2011). Very early rheumatoid arthritis is the major predictor of major outcomes: clinical ACR remission and radiographic non-progression. Ann Rheum Dis.

[19] Fransen J, van Riel PL (2005). The Disease Activity Score and the EULAR response criteria. Clin Exp Rheumatol.

[20] van Gestel AM, Prevoo ML, van’t Hof MA, van Rijswijk MH, van de Putte LB, van Riel PL (1996). Development and validation of the European League Against Rheumatism response criteria for rheumatoid arthritis: comparison with the preliminary American College of Rheumatology and the World Health Organization/International League Against Rheumatism Criteria. Arthritis Rheum.

[21] Prevoo ML, van Gestel AM, van T Hof MA, van Rijswijk MH, van de Putte LB, van Riel PL (1996). Remission in a prospective study of patients with rheumatoid arthritis: American Rheumatism Association preliminary remission criteria in relation to the disease activity score. Br J Rheumatol.

[22] Aletaha D, Smolen J (2005). The Simplified Disease Activity Index (SDAI) and the Clinical Disease Activity Index (CDAI): a review of their usefulness and validity in rheumatoid arthritis. Clin Exp Rheumatol.

[23] Sanz I, Wei C, Lee FE, Anolik J (2008). Phenotypic and functional heterogeneity of human memory B cells. Semin Immunol.

[24] Crespo M, Bosch F, Villamor N, Bellosillo B, Colomer D, Rozman M, Marcé S, López-Guillermo A, Campo E, Montserrat E (2003). ZAP-70 expression as a surrogate for immunoglobulin-variable-region mutations in chronic lymphocytic leukemia. N Engl J Med.

[25] Michelutti A, Gremese E, Morassi F, Petricca L, Arena V, Tolusso B, Alivernini S, Peluso G, Bosello SL, Ferraccioli G (2011). B cell subsets in the joint compartments of seropositive and seronegative RA and non-RA arthritides express memory markers and ZAP-70 and characterize the aggregate pattern irrespectively of the autoantibody status. Mol Med.

[26] Duty JA, Szodoray P, Zheng NY, Koelsch KA, Zhang Q, Swiatkowski M, Mathias M, Garman L, Helms C, Nakken B, Smith K, Farris AD, Wilson PC (2009). Functional anergy in a subpopulation of naive B cells from healthy humans that express autoreactive immunoglobulin receptors. J Exp Med.

[27] Kamburova EG, Koenen HJ, Boon L, Hilbrands LB, Joosten I (2012). In vitro effects of rituximab on the proliferation, activation and differentiation of human B cells. Am J Transplant.

[28] Wei C, Anolik J, Cappione A, Zheng B, Pugh-Bernard A, Brooks J, Lee EH, Milner EC, Sanz I (2007). A new population of cells lacking expression of CD27 represents a notable component of the B cell memory compartment in systemic lupus erythematosus. J Immunol.

[29] Moura RA, Cascão R, Perpétuo I, Canhão H, Vieira-Sousa E, Mourão AF, Rodrigues AM, Polido-Pereira J, Queiroz MV, Rosário HS, Souto-Carneiro MM, Graca L, Fonseca JE (2011). Cytokine pattern in very early rheumatoid arthritis favours B cell activation and survival. Rheumatology (Oxford).

[30] Kokkonen H, Söderström I, Rocklöv J, Hallmans G, Lejon K, Rantapää Dahlqvist S (2010). Up-regulation of cytokines and chemokines predates the onset of rheumatoid arthritis. Arthritis Rheum.

[31] Ferraccioli G, Bracci-Laudiero L, Alivernini S, Gremese E, Tolusso B, De Benedetti F (2010). Interleukin-1β and interleukin-6 in arthritis animal models: roles in the early phase of transition from acute to chronic inflammation and relevance for human rheumatoid arthritis. Mol Med.

[32] Gremese E, Tolusso B, Fedele AL, Canestri S, Alivernini S, Ferraccioli G (2012). Baseline ZAP-70+ B cell subset influences response to B-cell depletion therapy (BCDT) and early repopulation in Rheumatoid Arthritis. J Rheumatol.

