# Supplementary material for: Memory B cell subsets and plasmablasts are lower in early than in long-standing Rheumatoid Arthritis
Source: BMC Immunol. 2014 Sep 4;15:28. doi: 10.1186/s12865-014-0028-1 (PMC4168163; doi:10.1186/s12865-014-0028-1)
Supplement: Additional file 1: Table S1. — B cell subset distribution according to IgD and CD27 classification in patients with VERA, ERA and LSRA. [file 12865_2014_28_MOESM1_ESM.doc]

**Table S1.** B cell subset distribution according to IgD and CD27 classification in patients with VERA, ERA and LSRA

| **Variables** | **VERA** | **ERA** | **LSRA** | **Mann-Whitney test** | | |
| --- | --- | --- | --- | --- | --- | --- |
|  |  |  |  | ***p^a^*** | ***p^b^*** | ***p^c^*** |
| **N.** | **25** | **43** | **54** |  |  |  |
| **CD19+ (%)** | 10.1 ± 4.5 | 9.8 ± 4.2 | 6.9 ± 4.2 | *0.82* | *0.002* | *0.001* |
| **CD19+/IgD+CD27+ (%)** | 8.8 ± 8.3 | 11.1 ± 16.1 | 8.7 ± 6.5 | *1.00* | *0.68* | *0.54* |
| **CD19+/IgD+CD27- (%)** | 65.5 ± 14.8 | 62.5 ± 17.3 | 51.8 ± 23.1 | *0.84* | ***0.01*** | ***0.001*** |
| **CD19+/IgD-CD27+ (%)** | 16.7 ± 9.3 | 16.9 ± 8.1 | 22.2 ± 12.9 | *0.99* | *0.12* | *0.08* |
| **CD19+/IgD-CD27- (%)** | 7.3 ± 5.1 | 7.1 ± 4.0 | 12.3 ± 6.4 | *0.99* | ***0.001*** | ***<0.001*** |
| **CD19+/CD38+CD27+ (%)** | 2.0 ± 2.2 | 3.1 ± 5.0 | 7.6 ± 5.3 | *0.64* | ***<0.001*** | ***<0.001*** |
| **CD19+/ZAP-70+ (%)** | 4.6 ± 4.6 | 4.9 ± 5.3 | 4.9 ± 5.2 | *0.71* | *0.39* | *0.55* |

Data are represented as mean ± standard deviation. Values in bold are significant. VERA: very early rheumatoid arthritis; ERA: early rheumatoid arthritis; LSRA: long-standing rheumatoid arthritis. p^a^: Mann-Whitney test between VERA and ERA patients; p^b^: Mann-Whitney test between VERA and LSRA patients; p^c^: Mann-Whitney test between ERA and LSRA patients.
