# Supplementary figures and images for: Memory B cell subsets and plasmablasts are lower in early than in long-standing Rheumatoid Arthritis
Source: BMC Immunol. 2014 Sep 4;15:28. doi: 10.1186/s12865-014-0028-1 (PMC4168163; doi:10.1186/s12865-014-0028-1)

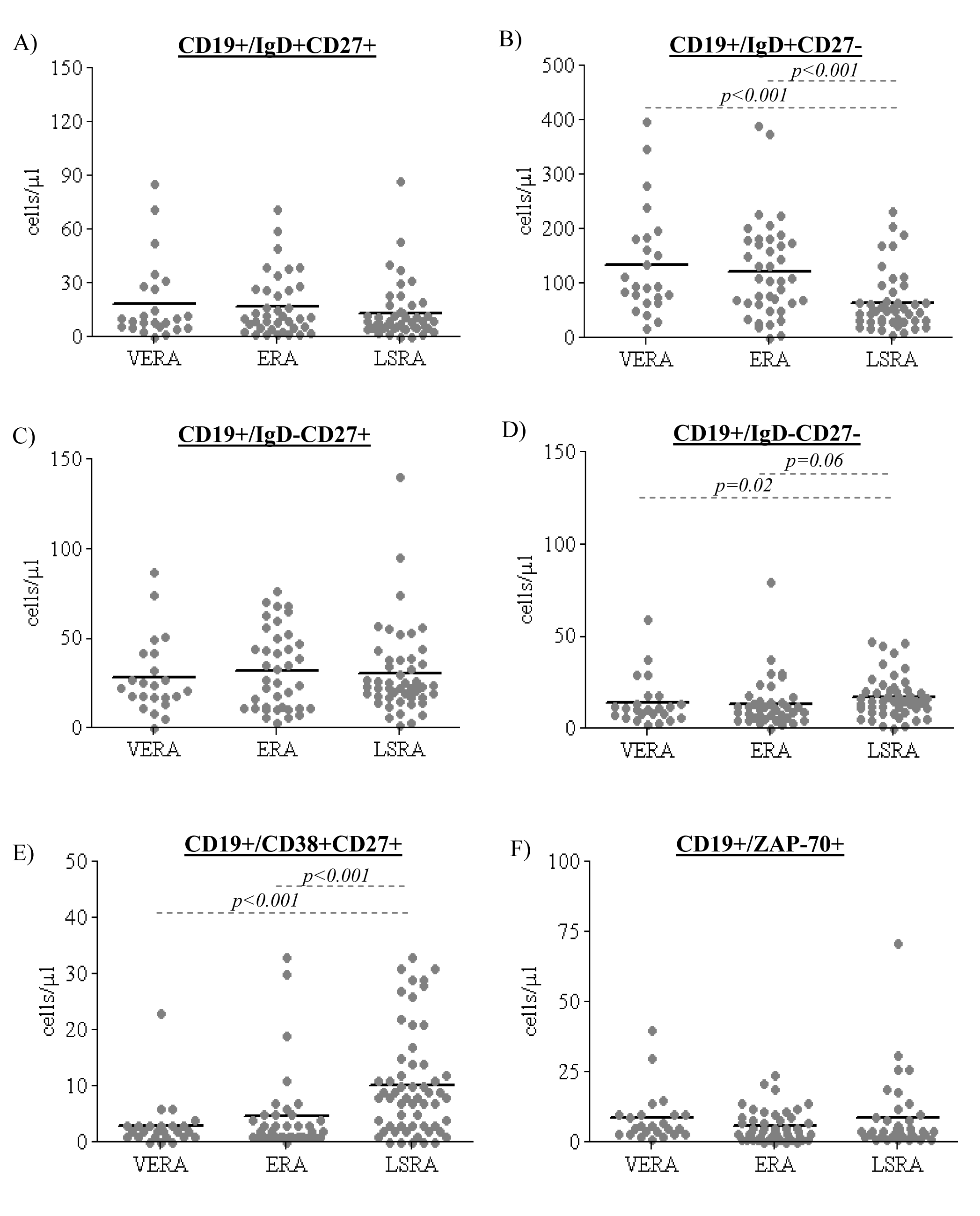

Supplement: Additional file 2: Figure S1. — Distribution of the absolute number of the different B cell subsets in patients with VERA, ERA and long standing-RA, according to the IgD and CD27 classification. Plots represent the absolute numbers of IgD + CD27+ cells (A), IgD + CD27- cells (B), IgD-CD27+ cells (C), IgD-CD27- cells (D), CD38 + CD27+ cells (E) and CD19+/ZAP-70+ (F) B cells. [file 12865_2014_28_MOESM2_ESM.tiff]

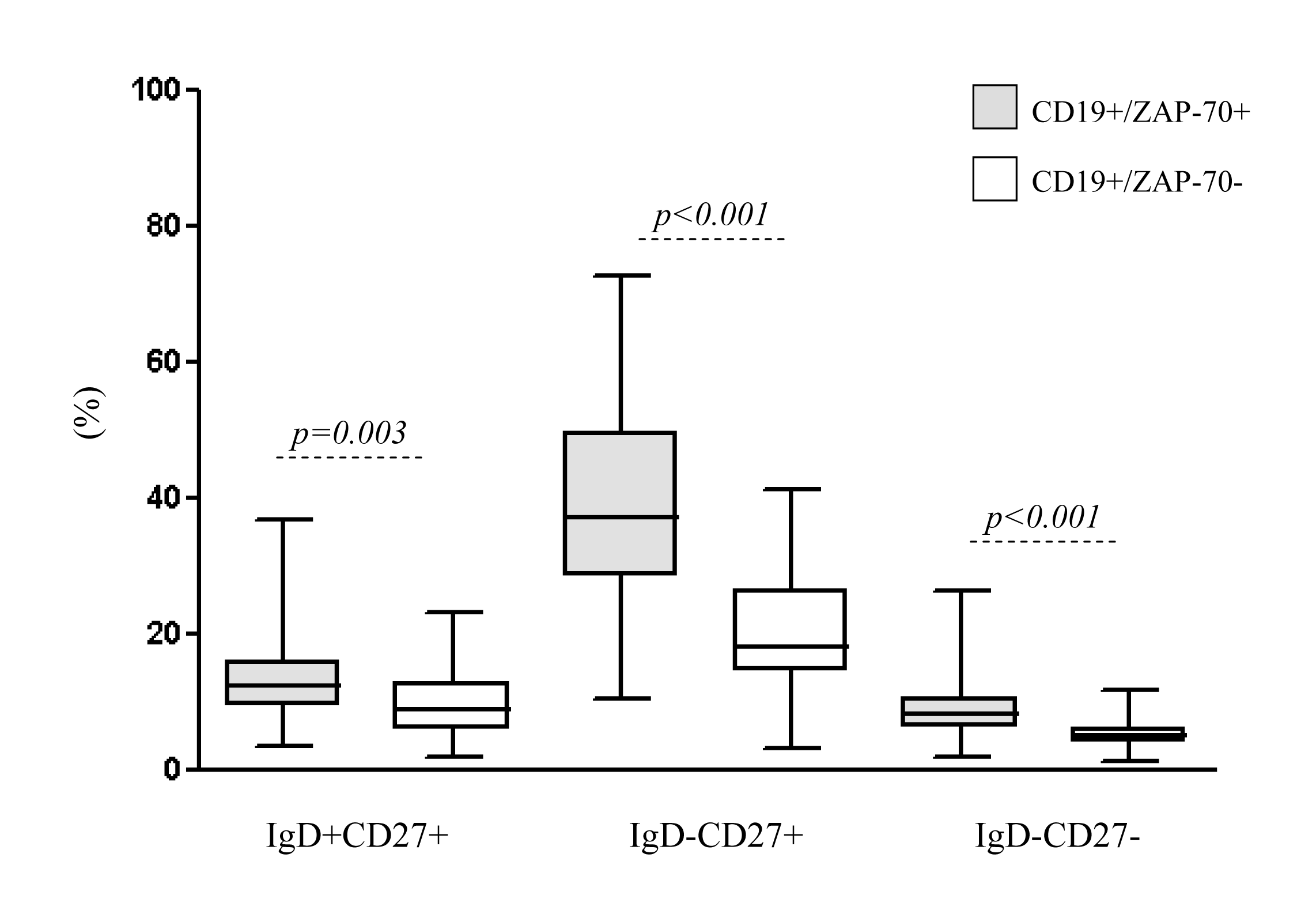

Supplement: Additional file 3: Figure S2. — Phenotypic characterization of ZAP-70+ and ZAP-70- B cells in VERA and ERA patients at baseline. The plot represents the percentage of IgD + CD27+ cells, IgD + CD27- cells, IgD-CD27+ and IgD-CD27- cells in CD19+/ZAP-70+ (grey) and in CD19+/ZAP-70- (white) cells. Box plot show the 10th, 25th, 50th (median), 75th and 90th percentiles of the variable. [file 12865_2014_28_MOESM3_ESM.tiff]
