# Supplementary material for: Memory B cell subsets and plasmablasts are lower in early than in long-standing Rheumatoid Arthritis
Source: BMC Immunol. 2014 Sep 4;15:28. doi: 10.1186/s12865-014-0028-1 (PMC4168163; doi:10.1186/s12865-014-0028-1)
Supplement: Additional file 4: Table S2. — Clinical and immunological parameters in patients with VERA and ERA, treated with conventional therapy, at the study entry and at 24 weeks follow-up visit. [file 12865_2014_28_MOESM4_ESM.doc]

**Table S2.** Clinical and immunological parameters in patients with VERA and ERA, treated with conventional therapy, at the study entry and at 24 weeks follow-up visit.

| **Variables** | **VERA-ERA**  **T0** | **VERA-ERA**  **T6** | **p** |
| --- | --- | --- | --- |
| **ESR (mm/1^hour)** | 38.0 ± 25.3 | 24.5 ± 21.5 | ***<0.001*** |
| **CRP (mg/l)** | 11.8 ± 17.3 | 5.2 ± 8.1 | ***0.01*** |
| **DAS** | 3.4 ± 1.1 | 1.9 ± 0.8 | ***<0.001*** |
| **BAFF (pg/ml)** | 897.7 ± 387.4 | 676.4 ± 255.2 | ***0.01*** |
| **IL-6 (pg/ml)** | 13.1 ± 19.3 | 3.3 ± 4.0 | ***0.004*** |
| **ACPA* (U/ml)** | 104.1 ± 63.9 | 80.5 ± 61.4 | *0.08* |
| **RF-IgM* (U/ml)** | 139.9 ± 149.2 | 58.0 ± 51.1 | ***0.001*** |
| **RF-IgA* (U/ml)** | 256.4 ± 393.0 | 56.7 ± 73.5 | ***0.02*** |

Data are represented as mean (S.D.). Values in bold are significant. * evaluated only on autoantibodies positive at the study entry.

ERA: early rheumatoid arthritis; ESR: erythrocyte sedimentation rate; CRP: C-reactive protein; DAS: disease activity score; BAFF: B-cell activating factor; ACPA: anti-citrullinated peptide antibodies; RF: rheumatoid factor.
